# Insights into the Function of the Unstructured N-Terminal Domain of Proteins 4.1R and 4.1G in Erythropoiesis

**DOI:** 10.1155/2011/943272

**Published:** 2011-08-28

**Authors:** Wataru Nunomura, Philippe Gascard, Yuichi Takakuwa

**Affiliations:** ^1^Department of Biochemistry, Tokyo Women's Medical University, Kawada 8-1, Shinjuku, Tokyo 162-8666, Japan; ^2^Department of Pathology, University of California, San Francisco (UCSF), San Francisco, CA 94143-0511, USA

## Abstract

Membrane skeletal protein 4.1R is the prototypical member of a family of four highly paralogous proteins that include 4.1G, 4.1N, and 4.1B. Two isoforms of 4.1R (4.1R^135^ and 4.1R^80^), as well as 4.1G, are expressed in erythroblasts during terminal differentiation, but only 4.1R^80^ is present in mature erythrocytes. One goal in the field is to better understand the complex regulation of cell type and isoform-specific expression of 4.1 proteins. To start answering these questions, we are studying in depth the important functions of 4.1 proteins in the organization and function of the membrane skeleton in erythrocytes. We have previously reported that the binding profiles of 4.1R^80^ and 4.1R^135^ to membrane proteins and calmodulin are very different despite the similar structure of the membrane-binding domain of 4.1G and 4.1R^135^. We have accumulated evidence for those differences being caused by the N-terminal 209 amino acids headpiece region (HP). Interestingly, the HP region is an unstructured domain. Here we present an overview of the differences and similarities between 4.1 isoforms and paralogs. We also discuss the biological significance of unstructured domains.

## 1. 4.1R in the Erythrocyte Membrane Skeleton

**Figure 1:**
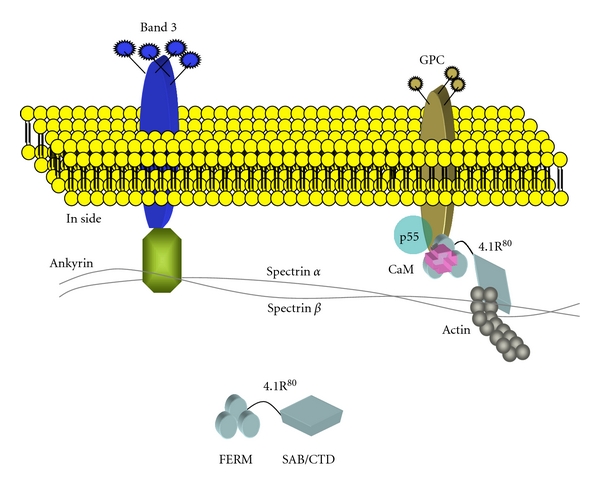
Structure of human erythrocyte membrane. Spectrin dimers underlying the membrane interact with transmembrane proteins band 3 through ankyrin and glycophorin C (GPC) through an actin complex and protein 4.1R^80^(4.1R^80^). 4.1R^80^ also forms a ternary complex with p55 and GPC. CaM binds to 4.1R^80^ in a Ca^2+^-independent manner.

The membrane skeleton, which underlies the erythrocyte plasma membrane, is made of a spectrin/actin lattice anchored to various transmembrane proteins via two specialized cytoskeletal proteins, 4.1R and red blood cell ankyrin, ankyrin-R [1]. 4.1R^80^ stabilizes horizontal interactions between spectrin heterodimers (*α*2-*β*2) and short actin (~14 molecules) filaments. Actin filaments interact with numerous accessory proteins, such as tropomyosin, myosin, tropomodulin, and adducin [1], which ensure reorganization of actin filaments. 4.1R^80^ interacts also with the transmembrane protein, glycophorin C (GPC) and with the membrane-associated guanylate kinase (MAGUK) protein p55, which also acts as an erythrocyte scaffolding protein (Figure 1). 

### 1.1. GPC

**Figure 2:**
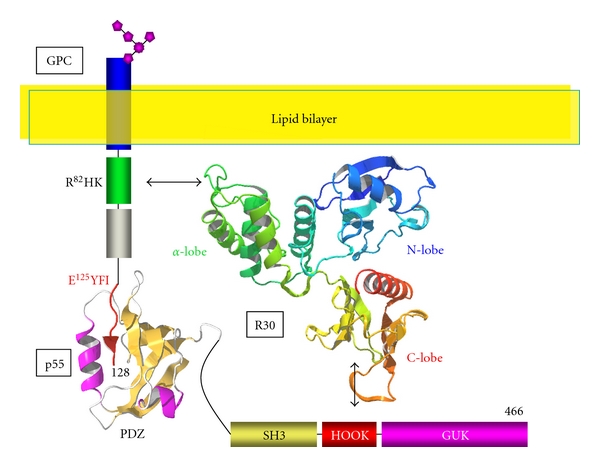
Organization of the R30/GPC/p55 ternary complex. The NMR structure of GPC peptide and PDZ domain complex has been previously reported [7] (PDB accession no. 2ejy). The HOOK domain is the 4.1R binding site for p55 [4].

GPC is 32 kDa single transmembrane protein expressed at ~50,000−100,000 molecules/erythrocyte. The cytoplasmic domain consists of 47 amino acids residues (ID: P04921). The R^82^HK sequence has been identified as the 4.1R binding sequence [2–4]. This RHK motif is highly conserved in the cytoplasmic domain of Neurexin IV, Paranodin, and TSLC1 (Tumor Suppressor Lung Cancer 1) [5]. Girault et al. have designated this RHK motif “GNP-motif,” after the single transmembrane 4.1R binding proteins, GPC, Neurexin IV, Paranodin [6]. GPC and other GNP-motif containing proteins possess a p55 binding motif, EYFI, in their C-terminal region (Figure 2). 

### 1.2. p55

p55 is a 55 kDa erythrocyte scaffolding protein that belongs to the membrane-associated guanylate kinase homologues (MAGUK) family (ID: Q00013). This protein is characterized by the presence of a PDZ (Postsynaptic density protein-95, Dlg (*Drosophila* disc large tumor suppressor), ZO-1 (Zonula Occludens-1)) domain, an SH (src-homology) 3 domain, and a catalytic inactive guanylate kinase like (GUK) domain, all of which function as protein-protein interaction modules (Figure 2) [8]. The number of p55 copies in the human erythrocytes is ~80,000. p55 is also called Membrane Palmitoylated Protein 1 (MPP1) since cysteine residues in the GUK domain can be palmitoylated [8]. However, there is still no direct evidence for the expression of palmitoylated p55 in living cells. Although the function of p55 in erythrocytes has not been clarified, p55 seems essential for maintenance of polarity in neutrophils [9] and in hair cells [10, 11]. Recently, NMR-based studies have enabled to characterize the 3D structural profile of the GPC peptide that interacts with the PDZ domain of p55 [7]. Mutational studies, based on the replacement of the phenylalanine residue in the EYFI motif with a cysteine residue (E^125^Y*C*I), have provided us with structural information on GPC binding to p55. Thus, 4.1R^80^ participates in the formation of two different ternary complexes in erythrocytes, the 4.1R^80^/GPC/p55/ complex and the 4.1R^80^/spectrin/actin complex. Ektacytometry studies have revealed that 4.1R plays a key role in controlling erythrocyte membrane mechanical properties. Indeed, resealed membranes prepared from erythrocytes totally or partially deficient in 4.1R^80^ show a dramatic decrease in membrane stability (reviewed in [12]). Interestingly, addition of either purified 4.1R^80^ or purified 10 kDa spectrin-actin binding domain of 4.1R^80^ to unstable 4.1R-deficient membranes is able to restore mechanical stability to such membranes. This demonstrates unequivocally an essential role for 4.1R^80^ and more specifically for a 21-amino-acid peptide encoded by exon 16 in the spectrin-actin binding domain, in maintaining membrane stability by promoting spectrin/actin interactions [1, 12]. 

### 1.3. Band 3

Membrane stability is also controlled in part by band 3-ankyrin-spectrin interaction (as shown in Figure 1). Band 3 is a 102 kDa 14-transmembrane protein which mediates exchange of HCO_3_^−^ and Cl^−^ and is therefore referred to as anion exchanger 1 (AE1) [1] (ID: P02730). It is expressed at 1,200,000 molecules/cell. It forms dimers that assemble into tetramers, each tetramer binding to one molecule of ankyrin. This is the base for the organization of the band 3-ankyrin-spectrin complex [13].

4.1R binds to the I^386^RRRY and L^343^RRRY sequences in band 3-cytoplasmic domain [14]. Although the crystal structure of the N-terminal cytoplasmic domain of band 3 has been reported, this structure is putative as the N-terminal 55 residues, including the L^343^RRRY sequence, were missing in the crystal [15]. The results indicate that band 3 has four 4.1R binding sites. The stoichiometry of band 3 binding to 4.1R is still unknown. The importance of band 3 in membrane architecture results from its role in anchoring the spectrin network through interaction with the scaffold protein ankyrin. We have demonstrated that 4.1R^80^ modulates band 3 interaction with ankyrin [16]. We have characterized a similar function for 4.1R^80^ in modulating ankyrin interaction with CD44, a single transmembrane protein which acts as receptor for hyaluronic acid [17].

The absence of 4.1R, ankyrin, or spectrin or selected mutations in these proteins result in alterations in erythrocyte shape and mechanical properties (reviewed in [1, 12]). We have demonstrated that 4.1R interacts with membrane protein analogues in zebrafish (*Danio rerio*) using *in vitro* binding assays [18, 19] (ID: NP_778259). Salomao et al. have documented that protein 4.1R^80^ can bind *in vitro* to additional erythrocyte transmembrane proteins, such as Kell, XK, Rh, and Duffy [20]. These interactions remain to be validated *in vivo*. The function of 4.1R has been inferred from the hematopoietic phenotype observed in human 4.1R-deficient patients, in transgenic 4.1R knock-out mice, and in zebrafish (*Danio rerio*) subjected to chemical mutagenesis [21]. 4.1R deficiency leads to hereditary elliptocytosis (HE), erythrocytes losing their typical biconcave disc shape to become elliptical. Thus, 4.1R acts in concert with other membrane proteins for maintaining normal erythrocyte shape [22].

## 2. PART I: 4.1R^80^ and 4.1R^135^ in Erythropoiesis

### 2.1. Overview of 4.1R Structure

4.1R forms multimolecular complexes with transmembrane proteins and membrane-associated proteins, such as spectrin and actin [1]. Such complexes, which are critical for maintaining structural stability in red blood cells, could well be involved in other functions in nonerythroid cells, such as, for example, signal transduction at sites of cell-cell and/or cell-matrix contacts.

4.1R^80^ (ID: P11171), present at approximately 200,000 copies per erythrocyte, can be extracted by high salt treatment of inside-out vesicles (IOVs), which correspond to erythrocytes membranes depleted of spectrin and actin. Based on its 622-amino-acid composition (reviewed in [1, 12]), the predicted molecular weight of 4.1R is only ~70 kDa, the discrepancy with the apparent molecular weight resulting in part from the unstructured domains of 4.1R. Limited *α*-chymotryptic digestion of 4.1R generates four polypeptides: a 30 kDa N-terminal membrane-binding domain, a 16 kDa domain, a 10 kDa spectrin-actin binding domain, and a 22/24 kDa C-terminal domain (reviewed in [1, 12]). A 4.1R isoform expressed in erythroblasts, but not in mature erythrocytes, contains an extra N-terminal 209 amino acids headpiece (HP) region. The apparent molecular weight of this 4.1R isoform in SDS-PAGE is ~135 kDa, and it is therefore referred to as 4.1R^135^. However, its theoretical molecular weight is ~100 kDa. This discrepancy results from the unstructured state of the HP region [23]. 

### 2.2. Unstructured N-Terminal and Structured 30 kDa FERM Domains of 4.1R^135^

**Figure 4:**
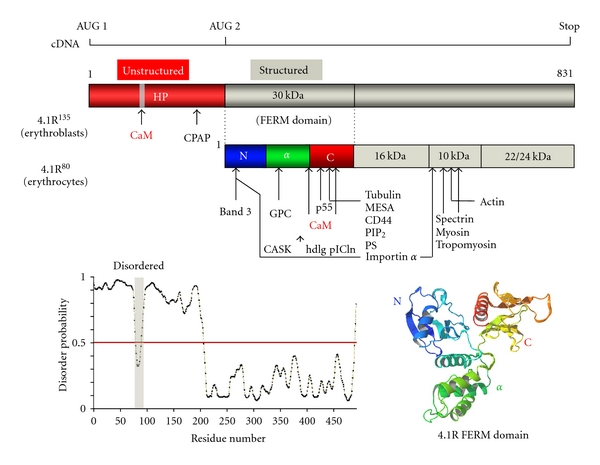
Primary structure of 4.1R isoforms and map of known binding partners for 4.1R. Translation of the prototypical red blood cell 80 kDa 4.1R isoform (4.1R^80^) is initiated at AUG-2, which is located in exon 4. Translation of the 135 kDa 4.1R isoform (4.1R^135^), an isoform expressed in early erythroblasts and other nucleated cells, is initiated at AUG-1, which is located in exon 2′ (ID: P11171). The 30 kDa membrane-binding domain is the so-called “FERM” domain. Disorder prediction for each domain has been established through the use of the PrDOS software package. An updated list of the binding partners identified for each domain of 4.1R is displayed. CPAP refers to a “*centrosomal protein 4.1R-associated protein*” reported by Hung et al. [24]. A 3D representation of the 30 kDa FERM domain of 4.1R, visualized with the MolFeat Ver. 4.6 software, is displayed (PDB accession no. 1GG3). The 30 kDa domain consists of three lobes (N-, *α*-, and C-lobe) and adopts a three-leaf clover shape [25].

We calculated the disorder probability of the N-terminal HP region and the FERM domain using the PrDOS software (http://prdos.hgc.jp/cgi-bin/top.cgi) [26]. A value greater than 0.5 reflects a disordered structure, with a probability of false prediction of 5% or less. Our analysis indicates a highly disordered structure for the HP region (amino acids 1–209) that contrasts with a highly ordered structure for the 30 kDa FERM domain (amino acids 210–507). Of particular note, while the overall 209aa HP region adopts a disordered structure, a short polypeptide (amino acids 70–80), corresponding to a previously identified Ca^2+^-dependent CaM-binding site [27, 28], does not (Figure 4). 

We experimentally demonstrated that the HP is an unfolded region by SDS-PAGE, size exclusion chromatography (SEC), and dynamic light scattering (DLS). The theoretical molecular weight of 4.1R HP (RHP) is 23 kDa but we estimate its apparent molecular weight as 55 kDa by SDS-PAGE [29]. Furthermore, SEC analysis reveals that RHP is eluted between IgG (150 kDa) and albumin (68 kDa) on a Sephacryl S-300 column. While the theoretical molecular weights of the proteins corresponding to amino acids 1–507 of 4.1R^135^ (RHP-R30) and to R30 (30 kDa FERM domain) are 56 kDa and 32 kDa, respectively, they migrate as polypeptides of >100 kDa and 35 kDa, respectively, on SDS-PAGE [29]. By DLS measurements, the hydrodynamic diameters of RHP, RHP-R30 and R30 are 7.6, 9.4, 5.6 nm, respectively (Nunomura, W., Shiba, K. and Takakuwa, Y., unpublished data). These hydrodynamic parameters enabled us to estimate the molecular weight of RHP, RHP-R30 and R30 to be 77, 127, and 40 kDa, respectively. The discrepancies between theoretical and apparent molecular weights for proteins containing RHP reflect the unfolded nature of this peptide.

In contrast, the consistency between theoretical and apparent molecular weights for R30 illustrates the folded nature of R30. Importantly, PrDOS-based analysis of full length 4.1R^135^ predicted the 30 kDa domain to be the only region in the whole protein to adopt an ordered (folded) structure. The crystal structure of 4.1R 30 kDa domain is reminiscent of the shape of a cloverleaf or of a propeller, with three clearly distinct lobes (PDB: 1GG3) [25]. First, the N-lobe, corresponding to the first 78 amino acids and which includes the band 3 binding motif L^37^EEDY, consists of 4 double-stranded *β*-strands. Second, the *α*-lobe, corresponding to the following 90 amino acids and which includes the GPC binding site, consists of 4 *α*-helices. Third, the COOH-terminal lobe (C-lobe), which contains the p55 binding surface, is made of seven *β*-strands, and ends with an *α*-helix (Figure 4). Although many membrane skeletal proteins contain intrinsically disordered (unfolded) regions, there are very few reports describing the function(s) of these intrinsically disordered region [30–35]. Our findings will contribute not only to a better understanding of the structure of membrane skeletal proteins but also of the function of intrinsically disordered proteins.

### 2.3. Expression of 4.1R^135^ and 4.1R^80^

In early stages of erythroblasts (CD34^+^ cells), 4.1R^135^ is the only isoform detected, 4.1R^80^ being completely absent. After the middle stage, which is reached after approximately 7 days in culture, expression of 4.1R^80^ increases dramatically. In mature erythrocytes, 4.1R^80^ predominates, 4.1R^135^ being hardly seen by immunocytochemical methods [29]. The complex mechanistic of 4.1R^135^-4.1R^80^ gene switching has been recently described by Parra et al. [36, 37]. 

### 2.4. Binding Profiles of 4.1R^135^ and 4.1R^80^ to Membrane Proteins and CaM Differ

Previous studies have shown that, while 4.1R^80^ binds to both band 3 and GPC in native inside-out vesicles (IOVs), it binds only to GPC in trypsinized IOVs [32]. Scatchard analysis indicates an apparent dissociation constant at equilibrium, *K*′, of 76 nM for 4.1R^80^ binding to trypsinized IOVs (i.e., to GPC). In contrast, *K*′ for 4.1R^80^ binding to native IOVs (i.e., to both GPC and band 3) reaches 340 nM. A similar analysis for 4.1R^135^ revealed that 4.1R^135^ binding to trypsinized IOVs (i.e., to GPC) is markedly weaker (*K*′ of ~2 *μ*M) than that of 4.1R^80^. In contrast, *K*′ for 4.1R^135^ binding to native IOVs is 230 nM, similar to that observed for 4.1R^80^. These findings imply that the presence or absence of HP in 4.1R isoforms modulates their binding affinity for GPC but not for band 3 [29]. 

In order to obtain independent confirmation of the binding affinities of 4.1R^135^ to band 3cyt and GPCcyt, we used the IAsys system based on the resonant mirror detection method [29]. In agreement with the binding data using IOVs described above, there was a dramatic difference in the binding affinity of 4.1R^135^ to band 3cyt and GPCcyt, the binding affinity being much higher for band 3cyt (23 ± 2 nM) than for GPCcyt (1327 ± 103 nM). In marked contrast, *K*_(*D*)_ values for binding of 4.1R^80^ to both band 3cyt and GPCcyt were very similar, in the submicromolar range. This confirmed an important role for HP in regulating 4.1R affinities for its two major transmembrane binding partners. In contrast to the marked differences in the binding affinities of 4.1R^135^ and 4.1R^80^ to band 3cyt and GPCcyt, the two isoforms bound to p55 with very similar affinities, in the submicromolar range. 

As expected from the data obtained with 4.1R^80^ and 4.1R^135^ isoforms, the addition of RHP to R30 (RHP-R30) results in a profound change in the ability of R30 to bind to band 3cyt and GPCcyt. Thus, the binding affinity of RHP-R30 for band 3cyt is 35-fold higher than for GPCcyt. Together, these findings highlight an important role for RHP in modulating the interaction of R30 with its two membrane-binding partners.

### 2.5. Differences in CaM Binding to 4.1R Isoforms

**Figure 5:**
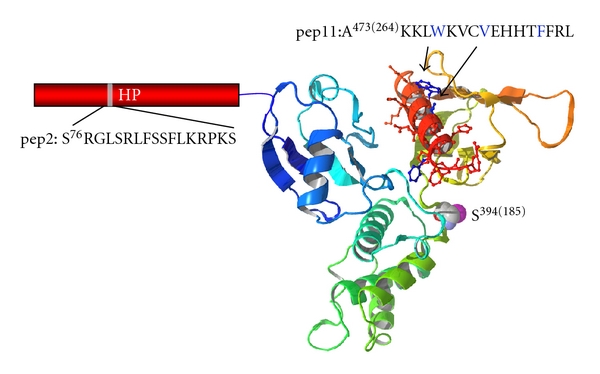
Mapping of the CaM-binding sites in 4.1R. 4.1R^135^ has three CaM-binding sites: pep2 in the HP region, S^185^ being the key residue for Ca^2+^-sensitive site, and Ca^2+^-independent sequence; pep9 and pep11 in the FERM domain. Numbers in parenthesis indicate amino acid numbering for 4.1R^80^ (AUG2 form in Figure 4).

We have previously documented that 4.1R^80^ binds to CaM with a *K*_(*D*)_ in the submicromolar range, both in the presence and absence of Ca^2+^ implying that this interaction is Ca^2+^-independent [38]. We have also examined the nature of the interaction between 4.1R^135^ and CaM. Kinetic analysis of 4.1R^135^ interaction with CaM using the IAsys system identified a very strong interaction with a *K*_(*D*)_ of 51 ± 5 nM in the presence of Ca^2+^. In the absence of Ca^2+^, the binding affinity decreased by over 100-fold. Thus, in contrast to 4.1R^80^, the interaction of 4.1R^135^ with CaM is strongly Ca^2+^ dependent. Probing of the HP region alone confirms a Ca^2+^-dependent interaction with CaM, implying that this region harbors the CaM-binding site [27, 28]. Our observations are in accordance with Leclerc and Vetter's study that identifies the S^76^RGLSRLFSSFLKRPKS peptide as the Ca^2+^-dependent CaM binding sequence in RHP [27, 28] (Figure 5). The stoichiometry of 4.1R^135^ binding to CaM in the presence of Ca^2+^ is 1 : 1 as assessed by the quartz crystal microbalance (QCM) method. These results indicate that Ca^2+^/CaM binds to the HP region but not to the 30 kDa domain [29]. 

### 2.6. Regulation of 4.1R^135^ Interactions with Membrane Proteins by Ca^2+^/CaM

**Figure 6:**
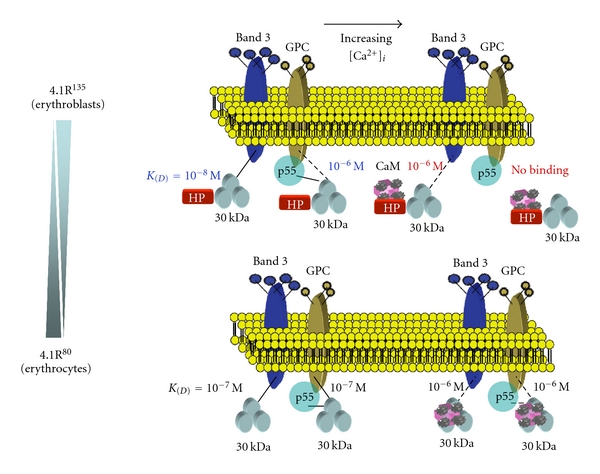
Model proposed for Ca^2+^/CaM-dependent regulation of 4.1R binding to membrane proteins. Erythroblast intracellular Ca^2+^ concentration is normally maintained at less than 0.1 *μ*M (10^−7^ M) [39, 40] (*upper panel*). At higher Ca^2+^ concentrations, CaM binds to the HP region. This results in a conformational and/or electric surface change which alters 4.1R binding sites, 4.1R^135^ interacting consequently with lower affinity with its binding partner band 3 and no longer interacting with GPC, and p55. This model implies a Ca^2+^/CaM-dependent regulation of protein 4.1R binding to transmembrane proteins. Erythrocyte intracellular Ca^2+^ concentration is normally maintained at less than 1.0 *μ*M (10^−6^ M) (lower panel). At this Ca^2+^ concentration, CaM is bound predominantly to the Ca^2+^-independent site located in peptide 11 of the 30 kDa domain (*see* [12, 38]). At higher Ca^2+^ concentrations, CaM-binding affinity for the Ca^2+^-dependent site, located in peptide 9 of the 30 kDa domain, increases. This results in a conformational and/or electric surface change which alters 4.1R binding sites, 4.1R interacting consequently with lower affinity with its binding partners p55, GPC, and spectrin/actin. This model implies that CaM regulates protein 4.1R binding to transmembrane proteins through Ca^2+^-independent and Ca^2+^-dependent binding sites.

**Figure 7:**
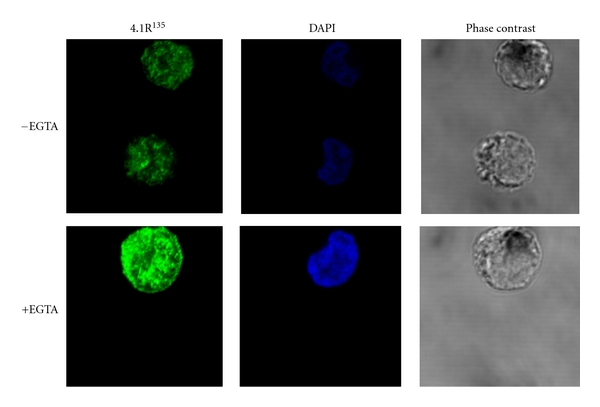
Effect of EGTA on the distribution of 4.1R^135^ in human erythroblasts (cultured for 7 days). Human erythroblasts were cultured in the presence or absence of 1 mM EGTA and immunostained with a rabbit antibody to RHP as previously described [29].

The binding affinity of 4.1R^135^ for band 3cyt is decreased by almost 2 orders of magnitude by Ca^2+^/CaM. Moreover, Ca^2+^/CaM completely abolishes the ability of 4.1R^135^ to bind to either GPCcyt or p55. Either 5 *μ*M CaM or 100 *μ*M Ca^2+^ alone has no affect on binding affinities. 4.1R^135^ binding to band 3cyt starts to decline at [Ca^2+^]_i_ greater than 10 nM (*p*Ca < 8) with a maximal inhibition at 100 *μ*M (*p*Ca > 4). Half maximal binding is observed at a [Ca^2+^]_i_ of 3.2 *μ*M (*p*Ca = 5.5). In the case of 4.1R^80^, Ca^2+^/CaM binding to the 30 kDa domain reduces about 10 times the binding affinity for band 3. Thus, we noted significant differences between 4.1R^135^ and 4.1R^80^ in the Ca^2+^-dependence for the binding of these two isoforms to CaM. In contrast to the Ca^2+^-independent binding of CaM to 4.1R^80^, CaM binding to 4.1R^135^ is strongly Ca^2+^ dependent. This difference is once again directly attributable to the HP region present in 4.1R^135^. Importantly, in contrast to band 3 and GPC that do not directly bind to the HP region, this region by itself binds to CaM in a Ca^2+^-dependent manner. Thus, it must be inferred that the CaM-binding site in the HP region is the dominant binding site for CaM in 4.1R^135^ and that this site prevents the binding of CaM to the Ca^2+^-independent binding site in 4.1R^80^. Furthermore, our finding that CaM dramatically decreases the binding of 4.1R^135^ to band 3 in a Ca^2+^-dependent manner and abolishes its binding to GPC and p55 has implications for the function of this 4.1R isoform in early erythroblasts. Indeed, while low levels of Ca^2+^ in early erythroblasts will lead to membrane association through high-affinity interaction with band 3, increasing levels of Ca^2+^ during erythroid differentiation will lead to the displacement of the protein from the membrane and to a possible degradation and loss of this isoform from erythroblasts. Our findings that, in early erythroblasts, a fraction of 4.1R^135^ is actually associated with the membrane lends support to this hypothesis [29] (Figure 6). Strikingly, in human erythroblasts cultured for 7 days and treated with 1mM EGTA, 4.1R^135^ is more clearly distributed at or near the plasma membrane than in nontreated cells (Figure 7). Precise quantitative measurements of Ca^2+^ levels in erythroblasts at different stages of maturation need to be performed to validate further this hypothesis.

## 3. PART II: 4.1R^135^ and 4.1G in Erythroblasts

4.1R^135^ and 4.1G are simultaneously expressed in erythroblasts and in nonerythroid cells, such as epithelial cells [42, 43]. The structure of the 30 kDa (FERM) domain of 4.1R and 4.1G is very similar. To date, there has not been any report about functional differences between 4.1R^135^ and 4.1G. We have shown for the first time differences in binding profiles of these two 4.1 proteins to membrane proteins. 

### 3.1. Structural Similarity between 4.1R^135^ and 4.1G

The primary amino acid sequence of the 30 kDa domain of 4.1G is 71% identical to that of 4.1R [42] (ID: O43491). 4.1G is therefore predicted to bind to many of the previously identified 4.1R binding partners. In contrast to the high conservation of the 30 kDa domain, the amino acid sequence identity of the HP region of 4.1G and 4.1R^135^ is quite low (35%). We therefore hypothesized that the HP region of 4.1R and 4.1G might regulate differently the binding properties of their respective 30 kDa domain. 

**Figure 8:**
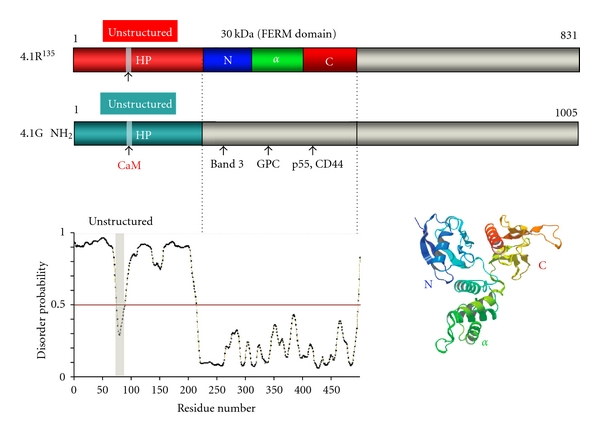
Primary structure of 4.1G. The primary structure of 4.1G resembles that of 4.1R^135^. *In vitro* binding assays show that the 30 kDa domain of 4.1G binds to previously characterized 4.1R binding partners. Modeling of 4.1G FERM domain 3D structure was performed *in silico *[41]. Spectrin and actin binding sites in 4.1G C-terminal domain are not displayed.

Computer analysis of the 3D structure of the 30 kDa domain of 4.1G has demonstrated that its folded clover-like structure is very similar to that of 4.1R [41] (Figure 8). This observation validates the structural basis for 4.1G binding to previously defined 4.1R binding partners through its 30 kDa domain. As observed for the 30 kDa domain of 4.1R, 4.1G could also interact with CaM in a Ca^2+^-independent manner. 

Using a combination of computational calculations (aimed at calculating the disorder probability based on PrDOS software analysis), SDS-PAGE analysis and size exclusion chromatography, we established that, like the HP region of 4.1R, the HP region of 4.1G adopts an unstructured state [41]. As expected from their similar structure, R30 and G30 are both folded polypeptides, this 30 kDa region representing the only structured (folded) domain for both proteins [41].

### 3.2. Expression of 4.1G and 4.1R^135^ in Erythroblasts

In erythroblasts, both 4.1G and 4.1R are expressed whereas the two other 4.1 gene products, 4.1B and 4.1N, are not (personal communication, Narla Mohandas, New York Blood Center). 4.1G is expressed after 7–12 days of culture as a ~70 kDa isoform containing the HP region. This suggests the occurrence of alternative splicing events targeting domains downstream of the HP region (FERM domain, spectrin-actin binding domain and/or C-terminal domain) in 4.1G. 

### 3.3. Differences in Binding Profiles of 4.1R^135^ and 4.1G to Membrane Proteins

4.1G binds to IOVs prepared from erythrocyte membranes. The apparent *K*′ values for 4.1G FERM domain (G30) and full length 4.1G binding to IOVs are 169 ± 67 nM and 207 ± 49 nM, respectively, as assessed by Scatchard plot analysis. These values are similar to those obtained using resonant mirror detection [41]. These findings demonstrate that 4.1G can bind to transmembrane proteins of the erythrocyte membrane through its 30 kDa domain.

4.1G interacts *in vitro* with band 3cyt and GPCcyt with *K*_(*D*)_s in the ~200 nM range. Importantly, the binding affinities of 4.1G for band 3cyt and GPCcyt are different from those of 4.1R^135^ despite the presence of an HP region in both proteins. Thus, 4.1G interacts with band 3cyt with a much lower affinity than 4.1R^135^, the reverse being observed for GPCcyt. These differences result mainly from differences in the association rate constant *k*_*a*_. In contrast, both 4.1G and 4.1R^135^ interact with p55 with similar affinities [44]. 

Binding affinities of full length 4.1G and of its 30 kDa domain (G30) for the membrane proteins described above are very similar, suggesting that 4.1G interacts with its binding partners primarily through G30, the headpiece GHP having a negligible effect on these interactions. This is in marked contrast to the interactions of the 30 kDa domain of 4.1R (R30) which are significantly affected by RHP [29]. Interestingly, recombinant chimera proteins consisting of either RHP and G30 (RHP-G30) or GHP and R30 (GHP-R30) showed similar binding affinities as G30 and R30. This implied significant differences in the structure and function of RHP and GHP. It should be noted that neither GHP nor RHP binds to any of these membrane proteins. 

We showed an important role for the HP region in regulating 4.1R^135^ 30 kDa domain binding to membrane proteins. Thus, the HP region improves accessibility of the N-lobe to band 3, but impairs accessibility of the *α*-lobe to GPC whereas it does not have a significant effect on the C-lobe [29]. 4.1G HP does not appear to modulate the accessibility of the three lobes in G30 to their respective binding partners, the binding profile of 4.1G being similar to that of G30. 

We demonstrated that 4.1G binds to various previously characterized 4.1R binding partners, including transmembrane proteins band 3, GPC, and p55, through its 30 kDa domain. The HP domain does not affect these interactions. However, Ca^2+^-dependent CaM binding to the HP region has a profound effect on the interaction of 4.1G with its binding partners. The documented binding profiles for 4.1G are markedly different from those previously reported for 4.1R^135^ [29]. Since the primary structure of the 30 kDa domain of 4.1G and 4.1R is highly conserved (71% sequence similarity), the differences in binding profiles are likely to arise primarily from the nonconserved HP region.

### 3.4. Similarities and Differences of CaM Binding to HP and 30 kDa Domains of 4.1R and 4.1G

Both full length 4.1G and GHP bind to a CaM Sepharose 4B column in the presence of Ca^2+^ and can be eluted with 5 mM EGTA. The *K*_(*D*)_ for CaM binding to 4.1G and GHP increases dramatically following chelation of Ca^2+^ with EGTA. These findings establish that CaM binds to 4.1G HP region in a Ca^2+^-dependent manner. These data recapitulate previous observations made for CaM binding to RHP and 4.1R^135^ [29]. The binding affinity of 4.1G to Ca^2+^/CaM is ~10 nM, and the stoichiometry is ~1 : 1 [41]. This observation strongly supports the importance of the HP region in mediating Ca^2+^-dependent CaM binding to 4.1G.

However, although CaM binds to the HP region of 4.1G in a Ca^2+^-dependent manner, it does not bind to 4.1G 30 kDa domain, as previously documented for 4.1R^80^ [38]. The HP region of 4.1G contains a sequence S^71^RGISRFIPPWLKKQKS that is 76% identical (13/17 residues) to the CaM-binding site in the HP region of 4.1R (S^76^RGLSRLFSSFLKRPKS) [27, 28]. Although the Ca^2+^-independent CaM-binding sequence previously identified in the 30 kDa domain of 4.1R^80^ is conserved in 4.1G [41], our results indicate that CaM binds to the HP region but not to the 30 kDa domain of 4.1G. It should be emphasized that although the HP by itself does not affect the binding of the 30 kDa domain of 4.1G to various membrane proteins, Ca^2+^/CaM binding to the HP markedly inhibits the ability of the 30 kDa domain of 4.1G to interact with its various binding partners. These findings have enabled us to document similarities and differences in the structural and functional properties of 4.1G and 4.1R^135^. 

### 3.5. Ca^2+^/CaM-Dependent Regulation of 4.1G Binding to Membrane Proteins

We have shown that Ca^2+^/CaM binding to the headpiece of 4.1G results in a complete inhibition of 4.1G binding to band 3cyt and p55 and in a significant increase in the *K*_(*D*)_ for 4.1G binding to GPCcyt. In light of the fact that CaM binds to the 30 kDa domain on 4.1R^80^ in the absence of Ca^2+^ and that CaM binding decreases the *K*_(*D*)_ of R30 for its binding partners in a Ca^2+^-dependent manner [17, 44], we examined the effect of CaM binding to G30 on its binding properties to membrane proteins using a RHP-G30 chimera protein. Binding affinities of RHP-G30 for band 3cyt, GPCcyt, p55, and CD44cyt were measured in the presence or absence of Ca^2+^ and CaM. The *K*_(*D*)_s obtained for each binding partner in the absence of CaM were similar to those obtained with full-length 4.1G. In contrast, binding assays performed with RHP-G30 preincubated with Ca^2+^/CaM showed a major decrease in binding affinity (7–10 fold in *K*_(*D*)_) of RHP-G30 for band 3cyt, GPCcyt, and p55. These results indicate that although CaM can bind to G30 independently of Ca^2+^, G30 interactions with membrane proteins can be regulated by CaM in a Ca^2+^-dependent manner. These results also indicate that the regulation of 30 kDa domain binding properties by unfolded HP domain has unique features in the case of 4.1R^135^ and 4.1G. 

**Figure 9:**
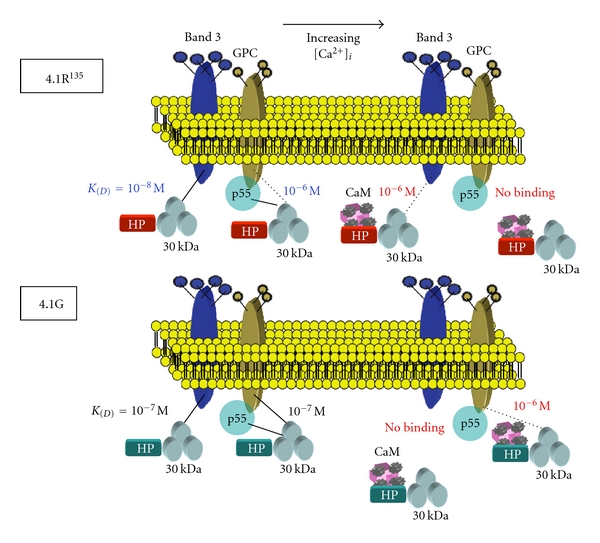
Model proposed for Ca^2+^/CaM-dependent regulation of 4.1G binding to membrane proteins. Erythroblast intracellular Ca^2+^ concentration is normally maintained at less than 0.1 *μ*M (10^−7^ M) as described in Figure 7 [39, 40]. 4.1G binds to band 3, GPC and p55 with a *K*_(*D*)_ of 10^−7^ M. At higher Ca^2+^ concentrations, CaM binds to the HP region with a *K*_(*D*)_ of 10^−8^ M. This results in a conformational and/or electric surface change which alters 4.1G binding sites, 4.1G interacting consequently with lower affinity with its binding partners GPC and no longer interacting with band 3 and p55. This model implies a Ca^2+^/CaM-dependent regulation of 4.1G binding to transmembrane proteins.

The Ca^2+^ concentration dependence of the CaM-modulated interaction of 4.1G with band 3cyt and GPCcyt has been demonstrated [38, 44]. The half maximal binding of 4.1R^135^ and 4.1G to band 3cyt and GPCcyt occurs at Ca^2+^ concentrations in the submicromolar range [39, 40], supporting the potential biological relevance of our biochemical findings [29]. Ca^2+^/CaM-dependent modulations of 4.1R^135^ and 4.1G binding to membrane proteins may be triggered upon signal transduction during erythroid development. Indeed, it has been documented that, at early stages of erythropoiesis, intracellular calcium levels increased from a basal level of 55 ± 5 nM to 259 ± 49 nM following binding of erythropoietin to its receptor [39]. Such an increase in intracellular calcium levels would be sufficient to modulate the interaction of 4.1R^135^ and 4.1G with its binding partners in erythroid cells. Our findings further suggest that 4.1G offers a unique opportunity to explore divergence of protein structure and function during evolution and development. In erythroblasts, we showed that, consistent with earlier reports [42, 43], 4.1G and 4.1R^135^ are both expressed during terminal erythroid differentiation and that both proteins can interact with common transmembrane proteins, such as band 3, GPC, and p55. Different binding affinities and Ca^2+^/CaM-dependent modulation of interaction with band 3 and GPC suggest that these 4.1 proteins may play specific roles in membrane biogenesis during terminal erythroid differentiation (Figure 9).

Thus, the unstructured HP domains of 4.1R and 4.1G seem to play a unique role in regulating the membrane-binding properties of those proteins. Understanding the structural basis for differences and similarities in 4.1 binding properties will help us unveil novel biological functions for various 4.1 gene products. To that end, we are currently carrying out a structural analysis of the HP-Ca^2+^/CaM complex using NMR and small-angle X-ray scattering (SAXS). These biophysical analyses should help us further understand the structural basis for the regulatory role of the unstructured HP domain.

## 4. Conclusion

During erythropoiesis, the HP domain acts as a regulator of 4.1R and 4.1G interaction with the plasma membrane. We hypothesize that these regulatory properties are in part the result of the unstructured conformation of the HP region. We also show that these regulatory properties depend on intracellular calcium concentrations, with these concentrations varying during erythropoiesis. Thus, the function of the HP domain may evolve depending on the structure of the 4.1 protein isoforms expressed at each stage of erythropoiesis. 

## 5. Future Studies on 4.1R^135^ and 4.1G

This paper focuses on the structure and function of the N-terminal intrinsically disordered region (HP) and membrane-binding FERM domain of 4.1R^135^ and 4.1G and on the role of Ca^2+^ in regulating binding to membrane proteins through CaM. Our findings are based on *in vitro* binding assays. Direct evidence for these interactions and their regulations in living cells remains to be established. Although it is known that the RHP contains phosphorylation sites [28, 45], the relationship between Ca^2+^/CaM regulation and phosphorylation remains to be investigated. 4.1G binds to spectrin/actin [46, 47] and receptors through its C-terminal region [48, 49]. Does Ca^2+^/CaM binding to HP also regulate these interactions? Answering such mechanistic questions will help us define the biological significances of 4.1R^135^ and 4.1G in the late stage of erythropoiesis.

## Figures and Tables

**Figure 3:**
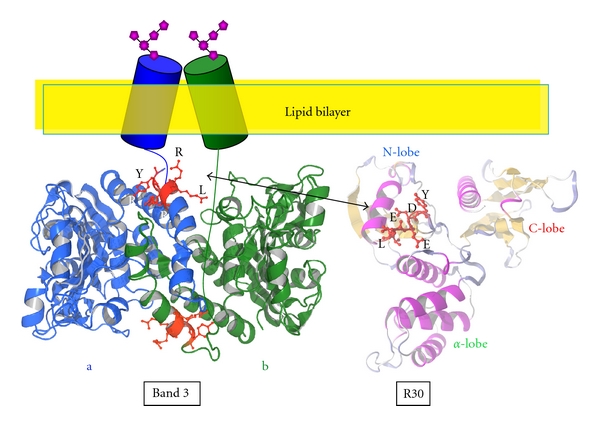
Characteristics of 4.1R interaction with band 3. Band 3 forms dimers in the membrane (*a* and *b*). The LRRRY sequence mediates interaction between monomers and is located in an *α*-helix. The band 3 binding sequence LEEDY, which mediates interaction with 4.1R, is located in the loop structure [15] (PDB accession no. 1hyn). There is no information about the stoichiometry of band 3 binding to 4.1R.

**Table 1:** 4.1R^135^ and 4.1G binding to membrane proteins.

Analyte	Ligand	*K*_*a*_ (M^−1^ s^−1^)	*K*_*d*_ (s^−1^)	*K*_*D*_ (nM)
4.1R^135^	band 3cyt	3.1 ± 0.2 × 10^5^	7.1 ± 0.2 × 10^−3^	23 ± 2
	GPCcyt	8.0 ± 0.2 × 10^3^	1.1 ± 0.1 × 10^−2^	1327 ± 103
4.1G	band 3cyt	8.0 ± 0.1 × 10^4^	1.4 ± 0.1 × 10^−2^	185 ± 23
	GPCcyt	5.6 ± 0.1 × 10^4^	8.1 ± 0.2 × 10^−3^	144 ± 5

**Table 2:** 4.1R^135^ and 4.1G binding to CaM.

Analyte	Ligand	Condition	*K*_*a*_ (M^−1^ s^−1^)	*K*_*d*_ (s^−1^)	*K*_*D*_ (nM)
4.1R^135^	CaM	EGTA	1.4 ± 0.2 × 10^3^	1.6 ± 0.1×10^−2^	11659 ± 2890
		Ca^2+^	2.0 ± 0.1 × 10^5^	1.5 ± 0.1×10^−2^	78 ± 10
4.1G	CaM	EGTA	3.7 ± 0.2 × 10^3^	8.3 ± 0.1 ×10^−3^	2245 ± 41
		Ca^2+^	9.4 ± 0.2 × 10^4^	5.1 ± 0.2 × 10^−3^	54 ± 3

## Appendix

Tables 1 and 2 summarize 4.1R^135^ and 4.1G binding kinetic parameters to the cytoplasmic tails of band 3 and GPC and to CaM in the presence or the absence of Ca^2+^. Although both 4.1R^135^ and 4.1G bind to Ca^2+^/CaM in the ~10^−8^ M *K*_(*D*)_ range, the *K*_(*D*)_ of 4.1R^135^ binding to CaM is 5 times higher than 4.1G in the absence of Ca^2+^. These results suggest that the binding profiles of 4.1R and 4.1G may differ in respect to CaM.

## Abbreviations

4.1G: Protein 4.1G
4.1R^80^: 80 kDa human erythrocyte protein 4.1
4.1R^135^: 135 kDa human erythroblast protein 4.1
Band 3cyt: Cytoplasmic domain of band 3
CaM: Calmodulin
CD44cyt: Cytoplasmic domain of CD44
FERM, Four-one: Ezrin, Radixin, Moesin
G30: 30 kDa membrane binding domain of protein 4.1G
GHP: Headpiece region of protein 4.1G
GHP-G30: Fusion protein of GHP and G30
GPC: Glycophorin C
GPCcyt: Cytoplasmic domain of GPC
HP: N-terminal head-piece region of 4.1 proteins
IOVs: Inside-out-vesicles of human erythrocytes
*K*_(*D*)_: Dissociation constant at equilibrium
R30: 30 kDa domain of protein 4.1R
RHP: Headpiece region of protein 4.1R
RHP-R30: Fusion protein of RHP and R30
RHP-G30: Chimera protein of RHP and G30.

## References

[1] Bennett V, Baines AJ (2001). Spectrin and ankyrin-based pathways: metazoan inventions for integrating cells into tissues. *Physiological Reviews*.

[2] Hemming NJ, Anstee DJ, Mawby WJ, Reid ME, Tanner MJA (1994). Localization of the protein 4.1-binding site on human erythrocyte glycophorins C and D. *Biochemical Journal*.

[3] Hemming NJ, Anstee DJ, Staricoff MA, Tanner MJA, Mohandas N (1995). Identification of the membrane attachment sites for protein 4.1 in the human erythrocyte. *Journal of Biological Chemistry*.

[4] Marfatia SM, Lue RA, Branton D, Chishti AH (1995). Identification of the protein 4.1 binding interface on glycophorin C and p55, a homologue of the *Drosophila* discs-large tumor suppressor protein. *Journal of Biological Chemistry*.

[5] Yageta M, Kuramochi M, Masuda M (2002). Direct association of TSLC1 and DAL-1, two distinct tumor suppressor proteins in lung cancer. *Cancer Research*.

[6] Girault JA, Labesse G, Mornon JP, Callebaut I (1998). Janus kinases and focal adhesion kinases play in the 4.1 band: a superfamily of band 4.1 domains important for cell structure and signal transduction. *Molecular Medicine*.

[7] Kusunoki H, Kohno T (2007). Structural insight into the interaction between the p55 PDZ domain and glycophorin C. *Biochemical and Biophysical Research Communications*.

[8] Dimitratos SD, Woods DF, Stathakis DG, Bryant PJ (1999). Signaling pathways are focused at specialized regions of the plasma membrane by scaffolding proteins of the MAGUK family. *BioEssays*.

[9] Quinn BJ, Welch EJ, Kim AC (2009). Erythrocyte scaffolding protein p55/MPP1 functions as an essential regulator of neutrophil polarity. *Proceedings of the National Academy of Sciences of the United States of America*.

[10] Mburu P, Kikkawa Y, Townsend S, Romero R, Yonekawa H, Brown SDM (2006). Whirlin complexes with p55 at the stereocilia tip during hair cell development. *Proceedings of the National Academy of Sciences of the United States of America*.

[11] Mburu P, Romero MR, Hilton H (2010). Gelsolin plays a role in the actin polymerization complex of hair cell stereocilia. *PLoS ONE*.

[12] Nunomura W, Takakuwa Y (2006). Regulation of protein 4.1R interactions with membrane proteins by Ca^2+^ and calmodulin. *Frontiers in Bioscience*.

[13] Michaely P, Bennett V (1995). The ANK repeats of erythrocyte ankyrin form two distinct but cooperative binding sites for the erythrocyte anion exchanger. *Journal of Biological Chemistry*.

[14] Jons T, Drenckhahn D (1992). Identification of the binding interface involved in linkage of cytoskeletal protein 4.1 to the erythrocyte anion exchanger. *EMBO Journal*.

[15] Zhang D, Kiyatkin A, Bolin JT, Low PS (2000). Crystallographic structure and functional interpretation of the cytoplasmic domain of erythrocyte membrane band 3. *Blood*.

[16] An XL, Takakuwa Y, Nunomura W, Manno S, Mohandas N (1996). Modulation of band 3-ankyrin interaction by protein 4.1: functional implications in regulation of erythrocyte membrane mechanical properties. *Journal of Biological Chemistry*.

[17] Nunomura W, Takakuwa Y, Tokimitsu R, Krauss SW, Kawashima M, Mohandas N (1997). Regulation of CD44-protein 4.1 interaction by Ca^2+^ and calmodulin. Implications for modulation of CD44-ankyrin interaction. *Journal of Biological Chemistry*.

[18] Nunomura W, Takakuwa Y, Cherr GN, Murata K (2007). Characterization of protein 4.1R in erythrocytes of zebrafish (*Danio rerio*): unique binding properties with transmembrane proteins and calmodulin. *Comparative Biochemistry and Physiology B*.

[19] Murata K, Nunomura W, Takakuwa Y, Cherr GN (2010). Two different unique cardiac isoforms of protein 4.1R in zebrafish, *Danio rerio*, and insights into their cardiac functions as related to their unique structures. *Development Growth and Differentiation*.

[20] Salomao M, Zhang X, Yang Y (2008). Protein 4.1R-dependent multiprotein complex: new insights into the structural organization of the red blood cell membrane. *Proceedings of the National Academy of Sciences of the United States of America*.

[21] Shafizadeh E, Paw BH, Foott H (2002). Characterization of zebrafish merlot/chablis as non-mammalian vertebrate models for severe congenital anemia due to protein 4.1 deficiency. *Development*.

[22] Takakuwa Y (2000). Protein 4.1, a multifunctional protein of the erythrocyte membrane skeleton: structure and functions in erythrocytes and nonerythroid cells. *International Journal of Hematology*.

[23] Diakowski W, Grzybek M, Sikorski AF (2006). Protein 4.1, a component of the erythrocyte membrane skeleton and its related homologue proteins forming the protein 4.1/FERM superfamily. *Folia Histochemica et Cytobiologica*.

[24] Hung LY, Tang CJC, Tang TK (2000). Protein 4.1 R-135 interacts with a novel centrosomal protein (CPAP) which is associated with the *γ*-tubulin complex. *Molecular and Cellular Biology*.

[25] Han BG, Nunomura W, Takakuwa Y, Mohandas N, Jap BK (2000). Protein 4.1R core domain structure and insights into regulation of cytoskeletal organization. *Nature Structural Biology*.

[26] Ishida T, Kinoshita K (2007). PrDOS: prediction of disordered protein regions from amino acid sequence. *Nucleic Acids Research*.

[27] Kelly GM, Zelus BD, Moon RT (1991). Identification of a calcium-dependent calmodulin-binding domain in Xenopus membrane skeleton protein 4.1. *Journal of Biological Chemistry*.

[28] Leclerc E, Vetter S (1998). Characterization of a calcium-dependent calmodulin-binding domain in the 135-kD human protein 4.1 isoform. *European Journal of Biochemistry*.

[29] Nunomura W, Parra M, Hebiguchi M, Sawada KI, Mohandas N, Takakuwa Y (2009). Marked difference in membrane-protein-binding properties of the two isoforms of protein 4.1R expressed at early and late stages of erythroid differentiation. *Biochemical Journal*.

[30] Eliezer D (2009). Biophysical characterization of intrinsically disordered proteins. *Current Opinion in Structural Biology*.

[31] Espinoza-Fonseca LM (2009). Reconciling binding mechanisms of intrinsically disordered proteins. *Biochemical and Biophysical Research Communications*.

[32] Dunker AK, Cortese MS, Romero P, Iakoucheva LM, Uversky VN (2005). Flexible nets: the roles of intrinsic disorder in protein interaction networks. *FEBS Journal*.

[33] Minezaki Y, Homma K, Nishikawa K (2007). Intrinsically disordered regions of human plasma membrane proteins preferentially occur in the cytoplasmic segment. *Journal of Molecular Biology*.

[34] Sigalov AB (2010). Membrane binding of intrinsically disordered proteins: critical importance of an appropriate membrane model. *Self/Nonself—Immune Recognition and Signaling*.

[35] Nørholm A, Hendus-Altenburger R, Bjerre G, Kjaergaard M, Pedersen SF, Kragelund BB (2011). The intracellular distal tail of the Na^+^/H^+^ exchanger NHE1 is intrinsically disordered: implications for NHE1 trafficking. *Biochemistry*.

[36] Parra MK, Gee S, Mohandas N, Conboy JG (2011). Efficient *in vivo* manipulation of alternative pre-mRNA splicing events using antisense morpholinos in mice. *The Journal of Biological Chemistry*.

[37] Parra MK, Tan JS, Mohandas N, Conboy JG (2008). Intrasplicing coordinates alternative first exons with alternative splicing in the protein 4.1R gene. *EMBO Journal*.

[38] Nunomura W, Takakuwa Y, Parra M, Conboy JG, Mohandas N (2000). Ca^2+^-dependent and Ca^2+^-independent calmodulin binding sites in erythrocyte protein 4.1. Implications for regulation of protein 4.1 interactions with transmembrane proteins. *Journal of Biological Chemistry*.

[39] Yelamarty RV, Miller BA, Scaduto RC, Yu FTS, Tillotson DL, Cheung JY (1990). Three-dimensional intracellular gradients in single human brust-forming units-erythroid-derived erythroblasts induced by erythropoietin. *The Journal of Clinical Investigation*.

[40] Miller BA, Scaduto RC, Tillotson DL, Botti J, Cheung JY (1988). Erythropoietin stimulates a rise in intracellular free calcium concentartion in single early human erythroid precursors. *The Journal of Clinical Investigation*.

[41] Nunomura W, Kinoshita K, Parra M (2010). Similarities and differences in the structure and function of 4.1G and 4.1R^135^, two protein 4.1 paralogues expressed in erythroid cells. *Biochemical Journal*.

[42] Parra M, Gascard P, Walensky LD, Snyder SH, Mohandas N, Conboy JG (1998). Cloning and characterization of 4.1G (EPB41l2), a new member of the skeletal protein 4.1 (EPB41) gene family. *Genomics*.

[43] Gascard P, Lee G, Coulombel L (1998). Characterization of multiple isoforms of protein 4.1R expressed during erythroid terminal differentiation. *Blood*.

[44] Nunomura W, Takakuwa Y, Parra M, Conboy J, Mohandas N (2000). Regulation of protein 4.1R, p55, and Glycophorin C ternary complex in human erythrocyte membrane. *Journal of Biological Chemistry*.

[45] Huang SC, Liu ES, Chan SH (2005). Mitotic regulation of protein 4.1R involves phosphorylation by cdc2 kinase. *Molecular Biology of the Cell*.

[46] Kontrogianni-Konstantopoulos A, Frye CS, Benz EJ, Huang SC (2001). The prototypical 4.1R-10-kDa domain and the 4.1G-10-kDa paralog mediate fodrin-actin complex formation. *Journal of Biological Chemistry*.

[47] Gimm JA, An X, Nunomura W, Mohandas N (2002). Functional characterization of spectrin-actin-binding domains in 4.1 family of proteins. *Biochemistry*.

[48] Lu D, Yan H, Othman T, Turner CP, Woolf T, Rivkees SA (2004). Cytoskeletal protein 4.1G binds to the third intracellular loop of the A1 adenosine receptor and inhibits receptor action. *Biochemical Journal*.

[49] Saito M, Sugai M, Katsushima Y, Yanagisawa T, Sukegawa J, Nakahata N (2005). Increase in cell-surface localization of parathyroid hormone receptor by cytoskeletal protein 4.1G. *Biochemical Journal*.

